# 

**Published:** 1908-04-25

**Authors:** 


### Resident Medical Officers' Department.

### NOTES, QUESTIONS, AND COMMENTS.

The editor of the Resident Medical Officers' Department of The Hospital invites contributions to this column both from resident medical officers and from other members of the profession. Short articles, notes, queries, or suggestions bearing upon this branch of medical practice will always receive careful consideration, and those that are suitable will be published in due course. Correspondence, short and to the point, is particularly invited, as by this means the value of the R.M.O. Department will be greatly increased.

Articles, whether in the form of letters to the editor or otherwise, should in no case exceed 600 words in length, and should, if possible, be shorter than this. Postcards with short notes, questions, or notifications of recent hospital appointments, will be welcomed.

All communications for this column must be guaranteed by the name of the writer, which will not be published unless he expressly wishes it, and should bear the words "R.M.O. Department" on the envelope or the address side of the postcard.

Items of news from the resident officers' quarters of the principal hospitals, infirmaries, and asylums throughout the kingdom are invited, and, when of sufficient general interest, will be published.

The editor will also be pleased to receive and reply to confidential communications from resident medical officers and to consider any suggestions that may be made for the improvement of this department of The Hospital.

Brief notes and comments on new methods of diagnosis and treatment which are under trial in hospitals and are likely to be of practical use to readers will be especially welcome; and we shall be glad to receive questions or comments bearing on these subjects both from general practitioners and from R.M. officers.

### Electricity and Gas from a Hygienic Standpoint.

The journal of the Royal Sanitary Institute for March contains a report by Dr. Samuel Rideal on an inquiry carried out by him for the purpose of determining and comparing the hygienic effects of gas and electricity, as used for ordinary domestic lighting. Dr. Rideal's observations were carried out in two rooms of similar construction, lighted respectively by gas burners and electric lamps of normal type, giving an equal illumination. The ventilation was restricted to an average of 2,000 cubic feet per hour. It was found that although gas combustion originally develops more heat than does electricity the average temperature of the rooms was practically the same under either illuminant, and the electric light did not show the superiority in coolness usually claimed. The increase of organic matter in the two rooms during the course of the evening was less with gas than with electric light, probably because the organic matter produced by persons is partially destroyed by the gas. Dr. Rideal concludes from his observations that carbonic acid *per se* has not the injurious effect which was formerly attributed to it, but considerable rises in the temperature and moisture content of a room, from whatever source, do have a prejudicial effect upon the well-being of the occupants, but even under the adverse conditions of ventilation purposely created for his inquiry, neither the temperature nor percentages of moisture in the rooms reached a point at which such effect could be detected by any of the recognised physiological tests.

